# Supplementary material for: HiCEnterprise: identifying long range chromosomal contacts in Hi-C data
Source: PeerJ. 2021 Apr 26;9:e10558. doi: 10.7717/peerj.10558 (PMC8083178; doi:10.7717/peerj.10558)
Supplement: Supplemental Information 1 [file peerj-09-10558-s001.pdf]

Supplementary Materials to the article entitled HiCEnterprise: Identifying long range chromosomal contacts in HiC data, written by Hania Kranas, Irina Tuszynska and Bartek Wilczynski.

Comparison of HiCEnterprise results with existing programs for prediction of long range interactions based on HiC maps

All files necessary to run the comparison described below are available at regulomics.mimuw.edu.pl/~irina/HiCEnterprise_VS_HiCCUP_Homer.

In order to compare HiCEnterprise with existing methods, we used HUVEC Hi-C maps that was kindly delivered by Henry Niskanen and described in our joined publication [1]. Hi-C maps are in the Homer program format (HUVEC_Henri) [2]. We tried to use HiC-DC [4], PSYCHIC [5], HICCUPS [6] from the Juicer package [3] and findTADsAndLoops.pl from Homer toolbox [2] to predict chromatin long range interaction based on Hi-C maps. However, we were unable to run both HiC-DC and PSYCHIC approaches. To run PSYCHIC a commercial Matlab package is needed with additional paid Statistics and Machine Learning Toolbox. During running the procedure of finding interactions of chromosome regions by the HiC-DC program that is written as an R package, we obtained an error that could indicate an incorrectly used function in R script. We used HiC-DC on MacOS version 10.14.6 and R version 3.6.1 as well as on Ubuntu 18.04 and R version 3.6.2.

We used the Homer tool [2] to produce Hi-C maps in hic format (HUVEC-TCC2-newRun-notx-filtered4.hic) that is required by HiCCUPS [6] approach.

```
> tagDir2hicFile.pl HUVEC_Henri -juicer auto -juicerExe "java -jar /mnt/work/Programs/Juicebox/juicer_tools_1.13.01.jar" -genome hg19 -p 6
```

Next we used the Juicer tool [3] as well as our script written in the python language (HiC_to_npy_converter.py) to convert hic format to npy that is acceptable by HiCEnterprise program. We created npy Hi-C map with a resolution of 25000 bp (hic_25kbp_KR/mtx-1-1.npy).

We run HiCCUPS on chromosome 1 Hi-C map with default parameters described in the example section of HiCCUPS web site (https://github.com/aidenlab/juicer/wiki/HiCCUPS#detailed-usage) with the option --ignore_sparsity as was suggested by the program during the first usage:

```
> java -jar juicer_tools_1.13.01.jar hiccups --cpu -m 500 -r 25000 -c 1 HUVEC-TCC2-newRun-notx-filtered4.hic HUVEC_HiCCUPS_25kbp_KR_chr1 --ignore_sparsity
```

We also run Homer script findTADsAndLoops.pl with appropriate parameters that are described by developer of this approach (http://homer.ucsd.edu/homer/interactions2/HiCTADsAndLoops.html):

> findTADsAndLoops.pl find ../HUVEC-TCC2-newRun-notx-filtered4 -cpu 6 -res 25000 -window 25000 -genome hg19 -maxDist 2500000 -minLoopDist 25000 -o NoBedregion_Res25k_wind25k_minLoop25K_mxDist2500k

We set the same values for the window, resolution, minLoopDist and maxDist options so that the results of the predictions of all methods are as similar as possible. We used window size 25000 because both HiCCUPS and HiCEnterprise estimate contacts between individual bins not between a set of bins of Hi-C maps, as Homer does. MinLoopDist was set to 25000 because other methods we are considering start calculating the probability of interaction from the first adjacent bin, while maxDist specifies the largest distance between chromatin loops, which in both Homer and HiCEnterprise methods was set to 100 bins.
Both HiCCUPS and Homer calculate only contacts between bins, so we can compare results of HiCCUPS (HUVEC_HiCCUPS_25kbp_KR_chr1/merged_loops.bedpe) and Homer (HUVEC_Homer/NoBedregion_Res25k_wind25k_minLoop25K_mxDist2500k.loop.2D.bed) with the part of HiCEnterprise responsible for interactions between regions.
Due to HiCEnterprise considers interactions of a bin with a set size of range of bins left and right from it (usually 100 both sides), we chose HiCCUP results that have anchors of the predicted loops closer than 101. Next we chose Homer results for chromosome 1, add them to bins found by HiCCUPS, remove duplicates and put them (396 regions) as an input file (joined_homer_hiccupKR_uniq.bed) of HiCEnterprise.

Next we run HiCEnterprise for chromosome 1 Hi-C map with the same resolution as HiCCUPS and Homer was run for:

> HiCEnterprise regions -c 1 -b 25000 -r joined_homer_hiccupKR_uniq.bed --hic_folders HUVEC_Henri -s HiCEnterprise_KR_stats -f HiCEnterprise_KR_figures --stat_formats bed -t 0.1 --plotting mpl

For each region of HiCEnterprise_KR_stats [6] we created a scatter plot with HiCEnterprise, Homer and HiCCUPS results (see Supplementary files HiCEnterprise_VS_HiCCUP_Homer_scatter.pdf). It shows that different types of HiCCUPS p-values indicate the same bin of interaction, therefore to clarify results we also created plots for q-value of Hi-CEnterprise and HiCCUP_V p-value (see Supplementary files HiCEnterprise_VS_HiCCUP_Homer_plot.pdf).

We show the results of overlapping HiCCUPS, HOMER and HiCEnterprise results in the paper. The overlap between methods is significant, despite very different methods to identify enriched regions, and HiCEnterprise is clearly the most sensitive of all methods.

In Figure S2, we can see that HiCEnterprise found an interaction between four regions, while both HiCCUPS and Homer found one contact, near each other and one of the HiCEnterprise results. As can be seen in Figure S2, results do not seem accidental and could be subject to detailed investigation. Even though at this point we cannot rule out the possibility could be an

artifact, stemming from potential genetic rearrangement of the region in the studied cell-line, it is a clearly enriched pair of regions and seems to be correctly detected as significant.

However, it should be emphasized that HiCEnterprise regions have anticipated contacts with almost all bins that HiCCUPS and Homer programs found and proposed much more statistically important and hypothetically interesting contacts (Figure 1 of the main paper).

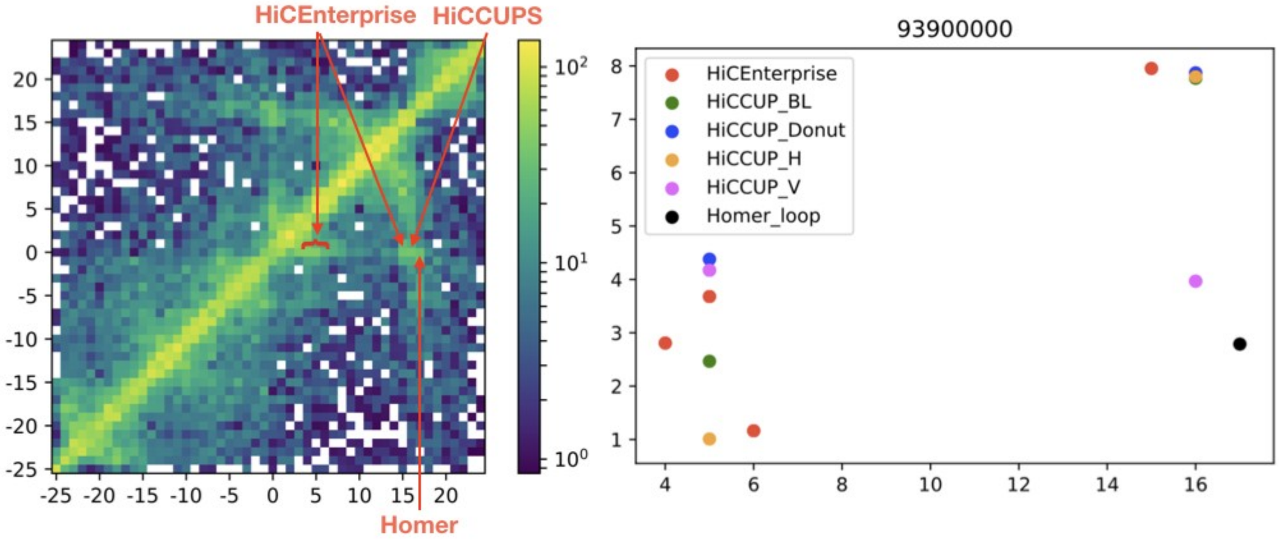

Figure S2. Left - a part of Hi-C map for chromosome 1 (3731-3781 bins) that illustrates regions that were identified by HiCEnterprise, Homer and HiCCUPS (marked red). Right - scatter plot with indicated bins and p-values for HiCEnterprise and HiCCUPS results for 3756 bin (indicated as 0 on the left part).

Based on our knowledge there are no freely available programs that predict interactions between domains so we were not able to compare the results of prediction inter- domains contacts of HiCEnterprise domain part with other methods. However, we calculated the probability of interactions between domains using HiCEnterprise domain method as well as identified the compartments to which the interacting domains belong.

First we have calculated domain borders using Sherpa algorithm (https://github.com/regulomics/sherpa) developed by our group, to run HiCEnterprise domain program. We have displayed the first 6 levels of sherpa domains to identify which domain boundary level best describes the domains whose long-range contacts can be seen in the Hi-C matrix (mtx-1-1.npy-sherpa_1chr_25kbp_HUVEC_6poz.out.pdf). We chose the fourth level as it matched best the expected size distribution of TADs.

Next we have changed the format of the domain boundary file to the format accepted by HiCEnterprise (domains_sherpa_1chr_25kbp_HUVEC.txt): domain_nr (starting from 1), chromosome_nr, start_of_domain (in bins), end_of_domain (in bins), sherpa_level (optional). Columns should be separated by a space.

Due to the scarcity of the HiC matrix for 25kbp resolution, we have used —all_domains option, to avoid removal of many potentially contacting domains. We have run HiCEnterprise domains program three times separately for each statistical tests, changing —distribution option to calculate inter-domains long range interactions using hypergeometric, poisson and negative binomial tests:

```
> HiCEnterprise domains -c 1 -b 25000 -s henri_stat_lvl4 -f henri_fig_lvl4 -m mtx-1-1.npy --
sherpa_lvl 4 -d domains_sherpa_1chr_25kbp_HUVEC.txt --distribution hypergeom --
all_domains --plotting
```

Then we used PCA to calculate compartments based on the Hi-C map. We wanted to understand if domains from one compartment interact with domains in the same compartment more often than expected by chance.

We have applied Principal Component Analysis (PCA) to the normalized interaction matrix and performed classification of each region of the chromosome with respect to the first component. The first main component (PC1) describes the “active” and “passive” chromatin compartments by adopting positive or negative values. Then we have calculated the number of positive or negative domains among those found by each method, as well as the number of positive-positive, positive-negative and negative-negative pairs of domains (Table S1). The domain has been classified as belonging to a positive or negative component based on the number of positive or negative PC1 values within the domain. If the number of positive and negative PC1 values in one domain is equal, the number of both positive and negative domains will increase by 0.5. Therefore, the number of domains in one range may not be an integer.

	**positive domains**	**negative domains**	**positive - positive obs/exp**	**positive - negative obs/exp**	**negative - negative obs/exp**
**Hypergeometric**	120.5	185.5	723/7260.125	1133/22352.75	1115/17205.125
**Poisson**	121.5	190.5	1169/7381.125	1152/23145.75	1745/18145.125
**Neg. binomial**	117	185	1047/6844.5	804/21645	1299/17112.5

Table S1: Affiliation of inter-domain interactions with active and passive compartments for all methods used to identify domain-domain interactions.

We have used chi-squared test to understand whether there is statistically significant difference between the expected frequencies of positive-positive, positive-negative and negative-negative domain pairs and observed values. We have calculated expected number of positive-positive and negative-negative pairs as (n**2)/2 (n means number of positive domains for positive-positive pairs or number of negative domains for negative-negative pairs). The expected number for positive-negative pairs was calculated as n*p, where n is the number of negative domains, while p is the number of positive domains.

For all methods, chi-square test rejected the null hypothesis according to which observed values had the expected frequencies (p-values< 10e-26, see Figure S3). Next we used binomial tests to check if the depletion of positive-negative pairs is statistically significant (Figure S3).For all used methods, the number of positive-negative pairs of interacted domains is much lower than expected, which is in line with our expectation, as it is known that domains from one compartment tend to interact with domains from the same compartment. It is worth to emphasize that in our analysis there are many single-bin domains, and the assignment of a single-bin domain to the appropriate compartment based on the PC1 component can often be affected by an error. This means that for chromosomes with larger domains, significant contacts between domains will occur even more often within one compartment.

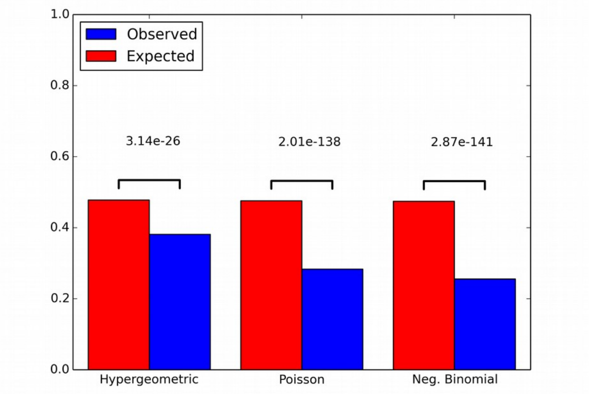

Figure S3: Statistical significance of the depletion of positive-negative domains pairs for all methods used for domains interaction calculation.

Enrichment of HiCEnterprise interactions in CTCF and cohesin

To see how confident we can be in the functionality of HiCEnterprise predictions, we decided to check how the interactions found by HiCEnterprise are enriched in CTCF and cohesin bound DNA regions. We used the HiCEnterprise interactions found for the comparison with other tools, as mentioned in the analysis above, and overlapped them with CTCF and RAD21 (cohesin component) Chip-seq peaks from HUVEC cells, provided by Henri Niskanen [1]. We only looked at the found interacting points by HiCEnterprise, and not the origins (lookout points to find interactions for). The intersection of interaction bins and CTCF/RAD21 peaks was performed with Bedtools Intersect, with the following command:

> **intersectBed -a <interaction_bins_bed> -b <peak_bins_bed> -u -wa -sorted**

on presorted bed files generated for both interactions and peak data. After, a simple count of unique interaction regions found to have an overlap with the peaks was calculated. As a background to compare to, we took all the bins queried by the program for possible interaction (all located +/- 100 bins from an origin/lookout point), constrained by the chromosome sizes and performed the same overlap analysis for them. Counts can be found in Table 2.

	**# bins**	**# (%) CTCF overlaps**	**# (%) RAD21 overlaps**
**HiCE unique**	893	499 (55.88%)	406 (45.46%)
**HiCE common**	362	285 (78.73%)	256 (70.72%)
**HiCE total**	1255	784 (62.47%)	662 (52.75%)
**all queried bins**	77034	32981 (42.81%)	24708 (32.07%)
**queried bins deduplicated**	9081	3403 (37.47%)	2553 (28.11%)

Table 2. *Interacting bins for HiCEnterprise (HiCE) and all queried by the tool with the corresponding number and percentage of them overlapping CTCF or RAD21 bound bins.*

HiCEnterprise interactions are enriched in CTCF and RAD21 bound DNA regions over the background of all queried possible interacting regions. To check if the comparisons are statistically significant, we performed some binomial tests using scipy.stats python package
>>>
scipy.stats.binom_test(HiCE_peak_number,HICE_bins_number,queried_bins_peak_percentage)
and found our enrichments to be statistically significant, regardless of whether compared to all queried bins or deduplicated, as can be seen in Table 3.

**HiCE dataset**	**Peaks set**	**queried bins version**	**p-value**
total	CTCF	all	1.85*10^-44
total	CTCF	deduplicated	1.15*10^-71
total	RAD21	all	1.52*10^-51
total	RAD21	deduplicated	8.27*10^-75

Table 3. *P-values from binomial tests for enrichments of HiCE interactions in CTCF/RAD21 peaks over the background of all queried bins*

To summarize, HiCEnterprise interactions are significantly enriched in CTCF/RAD21 bound regions. Common interactions (found by HiCEnterprise and one of the other tools: HOMER, HiCCUPS) are more enriched than the ones found uniquely by HiCEnterprise, while the unique ones are still enriched significantly over the background.

Clusters of HiCEnterprise interactions

As stratification of the genome into equal-sized bins is quite a rigid one, sometimes one might expect that an important interacting region might fall in-between two such bins. Moreover, it is interesting to see how often the interaction signal might “spill” into neighboring bins, or when we have a very long region intensely interacting with our point of interest. To study that, we simply checked how often, for each origin, its predicted interactions come from neighboring bins, and how many of them are consecutive. For the total of 1446 unique origin-interaction bins found, after clustering, we obtain 849 “long” interactions. Those consist of 525 of length 1, 180 of length 2, 78 of length 3, 37 of length 4, 14 of length 5, 5 of length 6, 4 of length 7, 3 of length 8, and 3 of length 9 bins. Out of those 849 “long” interactions, 481 consist just of unique HiCEnterprise-found interaction bins (382 of length 1, 74 of length 2, 16 of length 3, 7 of length 4, 2 of length 5 bins), 150 consist just of common interaction bins, found by both HiCEnterprise and other tools (143 of length 1, 7 of length 2) and 218 are mixed - unique and common (99 of length 2, 62 of length 3, 30 of length 4, 12 of length 5, 5 of length 6, 4 of length 7, 3 of length 8, 3 of length 9). To summarize, while most of the interactions are constrained to 1-3 bins, probably with the interacting regions lying somewhere in-between those, we also find very little of some very long interactions - up to 9 bins. Some of the possible reasons for that might be an overall activity or chromatin condensation in the wider region, as those very long interactions are found together with interactions found by other tools.

References

1. Niskanen, H., Tuszynska, I., Zaborowski, R., Heinaniemi, M., Yla-Herttuala, S., Wilczynski, B., and Kaikkonen, M. U. Endothelial cell differentiation is encompassed by changes in long range interactions between inactive chromatin regions. Nucleic acids research. NAR, February 2018; 46, 4
2. Heinz S, Benner C, Spann N, Bertolino E et al. Simple Combinations of Lineage-Determining Transcription Factors Prime cis-Regulatory Elements Required for Macrophage and B Cell Identities. Mol Cell 2010 May 28;38(4):576-589
3. Durand N.C., Shamim M.S, Machol I., Rao S.S.P., Huntley M.H., Lander E.S., Lieberman Aiden E.. Juicer provides a one-click system for analyzing loop-resolution Hi-C experiments. Cell Systems, 2016; 3(1).

4. Carty M., Zamparo L., Sahin M., Gonzalez A., Pelossof R., Elemento O., Leslie CS. An integrated model for detecting significant chromatin interactions from high-resolution Hi-C data. *Nat. Commun.* 8, 15454 doi: 10.1038/ncomms15454 (2017).
5. Ron G., Globerson Y., Moran D., Kaplan T. Promoter-enhancer interactions identified from Hi-C data using probabilistic models and hierarchical topological domains. *Nat. Commun.* 2017; 8: 2237
6. Rao SS, Huntley MH, Durand NC, Stamenova EK, Bochkov ID, Robinson JT, Sanborn AL, Machol I, Omer AD, Lander ES, Aiden EL. A 3D map of the human genome at kilobase resolution reveals principles of chromatin looping. Cell. 2014 Dec 18;159(7):1665-80
7. http://regulomics.mimuw.edu.pl/~irina/HiCEnterprise_VS_HiCCUP/HiCEnterprise_stats_nan/regions_hiccup-hic_forHiCEnterprise-significant1-100x25000bp-0_1.be
